# Colonoscopy reduces colorectal cancer mortality: A multicenter, long-term, colonoscopy-based cohort study

**DOI:** 10.1371/journal.pone.0185294

**Published:** 2017-09-28

**Authors:** Ryota Niikura, Yoshihiro Hirata, Nobumi Suzuki, Atsuo Yamada, Yoku Hayakawa, Hirobumi Suzuki, Shinzo Yamamoto, Ryo Nakata, Junko Komatsu, Makoto Okamoto, Makoto Kodaira, Tomohiro Shinozaki, Mitsuhiro Fujishiro, Toshiaki Watanabe, Kazuhiko Koike

**Affiliations:** 1 Department of Gastroenterology, Graduate School of Medicine, The University of Tokyo, Tokyo, Japan; 2 Division of Gastroenterology, The Institute for Adult Diseases, Asahi Life Foundation, Tokyo, Japan; 3 Department of Gastroenterology, Japanese Red Cross Medical Center, Tokyo, Japan; 4 Department of Health Care, Japanese Red Cross Medical Center, Tokyo, Japan; 5 Department of Gastroenterology, JR Tokyo General Hospital, Tokyo, Japan; 6 Department of Gastroenterology, Yaizu City Hospital, Shizuoka, Japan; 7 Department of Biostatistics, School of Public Health, The University of Tokyo, Tokyo, Japan; 8 Endoscopy and Endoscopic Surgery, The University of Tokyo Hospital, Tokyo, Japan; 9 Deapartement of Surgical Oncology and Vascular Surgery, Graduate School of Medicine, The University of Tokyo, Tokyo, Japan; University Hospital Llandough, UNITED KINGDOM

## Abstract

**Background:**

There are limited colonoscopy-based cohort data concerning the effectiveness of colonoscopy in reducing colorectal cancer deaths. The aim of this study was to clarify whether colonoscopy reduces colorectal cancer mortality.

**Methods:**

A cohort of 18,816 patients who underwent colonoscopy without a diagnosis of colorectal cancer between 2001 and 2010 at high colonoscopy procedure volume centers was selected. Patient characteristics and colonoscopy findings were assessed. The main endpoint was colorectal cancer death (all, right-sided, and left-sided cancers), and data were censored at the time of the final visit or the final colonoscopy. The standardized all colorectal, colon, and rectal cancer mortality rates were estimated with reference to those of the general Japanese population. Additional outcome was all- cause death and the standardized all-cause mortality rate was also estimated.

**Results:**

The total observed person-year mortality for colorectal cancer was 67,119. Of these, 4, 3, and 1 patients died from colorectal, colon, and rectal cancers, respectively; these values were significantly lower than the number of expected deaths in the general population, estimated to be 53.1, 34.0, and 19.1, respectively. The standardized mortalities for all colorectal, colon, and rectal cancers were 0.08 (95% confidence interval (CI), 0.02–0.17), 0.09 (95% CI, 0.02–0.22), and 0.05 (95% CI, 0.0002–0.21), respectively. There were 586 all-cause deaths (3.11%) during the observation period. The standardized all-cause mortality ratios were 0.22 (95% CI, 0.206–0.23).

**Conclusions:**

The colorectal cancer mortality of patients who received colonoscopy without colorectal cancer diagnosis decreased significantly compared with that of individuals in the general population. These results were compatible even in patients with right-sided colon cancer.

## Introduction

Colorectal cancer is a potentially preventable or curable disease. However it causes approximately 38.7 deaths per 100,000 population annually in Japan [1] and that mortality rate is higher than that reported in the United States (14.4 deaths per 100,000 populations) [2]. Preventing CRC is a very important public health issue in Japan. Colonoscopy reportedly reduces colorectal cancer incidence in patients who have a negative result of an initial colonoscopy.[3–12] However, there is concern whether the protective effect of colonoscopy extends to colorectal cancer mortality after a colonoscopy.

Recently, several case-control and cohort studies using population-based data have reported that colonoscopy potentially decreases colorectal cancer mortality[13–16] in cases of left-sided colon cancer.[17,18] However, little evidence exists regarding the protective effect of colonoscopy on colon cancer mortality as a primary outcome, including the right-sided colon, especially in longitudinal study using colonoscopy-based cohort data. Therefore, further studies are required to investigate the protective effect of colonoscopy on colorectal cancer mortality.

To address these issues, we performed a retrospective multicenter colonoscopy-based cohort study. The aim of this study was to clarify the effect of colonoscopy on colorectal cancer mortality and incidence after colonoscopy by comparing patients who received negative colonoscopy with the general population.

## Materials and methods

### Study design and setting

We performed a retrospective cohort study using an electronic colonoscopy database and electronic clinical charts from the colorectal cancer surveillance database between 2001 and 2010. The database included data from one referral hospital and four tertiary hospitals with high colonoscopy procedure volume. The following information was collected: patient characteristics, indications for colonoscopy, and colonoscopy findings. In colorectal cancer cases, the site of cancer, the Union for International Cancer Control cancer stage, and the therapy were also recorded. Comorbidities were recorded according to the International Classification of Diseases and Related Health Problems 10^th^ revision codes.[19] The end of follow-up of the cohort was December 2015. The lost to follow-up was defined as final visit after colonoscopy examination.

### Participants

We included patients who received a colonoscopy between 2001 and 2010. The indications for colonoscopy were as follows: i) asymptomatic screening, ii) post-polypectomy surveillance, and iii) a positive FIT. We systematically excluded patients aged < 20 years, those with a previous diagnosis of colorectal cancer or inflammatory bowel disease. We also excluded patients who received colonoscopy with a colorectal cancer diagnosis within 6 month as to exclude procedures intended to diagnose colorectal cancer in S1 Fig (because these could have potentially biased the association between colonoscopy and cancer death and incidence). These eligible criteria were similar to previous studies.[16, 20–24] This study was approved by the Institutional Review Board of The University of Tokyo (No. 2058).

### Colonoscopy examination

Colonoscopy examinations were performed principally using an electronic video endoscope (Olympus Optical, Tokyo, Japan) by a gastroenterologist, with full bowel preparation using a polyethylene glycol solution. All colonoscopy diagnoses were immediately recorded in the electronic endoscopic database after colonoscopy examination.

Follow-up or surveillance colonoscopies were performed based mainly on the Japanese clinical practice guidelines for management of colorectal polyps,[25] and the timing differed according to the attending physician.

### Outcomes and variables

The primary outcome was colorectal cancer death (all colorectal, right-sided colon, and left-sided colon cancer death), and all colorectal cancer death was identified based on a death certificate or clinical charts. Right-sided colon cancer included the cecum, ascending, and transverse colon cancer, and left-sided colon cancer included the descending, sigmoid, and rectal cancer. The primary endpoint (colorectal cancer death) was censored at the date of the final visit. The secondary outcome was colorectal cancer incidence (all colorectal, right-sided colon, and left-sided colon cancer incidence). Secondary endpoint (colorectal cancer incidence) was censored at the date of the final colonoscopy. All colorectal cancer cases were diagnosed by colonoscopy and were confirmed by a pathological examination. An additional outcome was all-cause death. An additional endpoint (all-cause death) was censored at the date of the final visit.

We evaluated the patients’ age, sex, family history of colorectal cancer, comorbidities, smoking status, FIT results, polyp size, therapeutic colonoscopy including polypectomy, endoscopic mucosal resection and endoscopic submucosal dissection, and the number of colonoscopies performed. Age was categorized into quintiles.

### Comparison group: General population

To compare the colorectal, colon, and rectal cancer mortality rates and incidences in our study to those of the Japanese general population, we estimated the standardized mortality and incidence ratios. The colorectal cancer included the cecum to rectal cancer; the colon cancer included cecum to sigmoid colon cancer. The expected numbers of colorectal cancer deaths and incidences were determined using age- and sex-specific data on the mortality rate and incidence of colorectal cancer in the general Japanese population, provided by the Center for Cancer Control and Information Services of the National Cancer Center in Japan.[26] The National Cancer Center established a database of colorectal cancer incidence and mortality for the entire Japanese population in 1958 and revised the data annually.

### Statistical analysis

Colorectal cancer mortality and incidence, and all-cause mortality are presented in person-years. The standardized mortality and incidence ratios were calculated as the ratio of the observed to the expected number of patients who died from or developed, respectively, colorectal cancer (all colorectal, colon, and rectal cancer). The 95% confidence intervals (CI) were estimated assuming a Poisson distribution following a variance-stabilizing transformation.

The Kaplan-Meier estimate of the cumulative probability over a 10-year period was calculated. Univariate and multivariate Cox proportional hazard models were used to estimate hazard ratios (HRs) with 95% CIs. The multivariate analysis was adjusted for age and sex. Missing family history data (*n* = 15,251), current smoking status (*n* = 9,617), 10-mm adenoma detection (*n* = 5,303), and 20-mm adenoma detection (*n* = 5,303) were imputed using the fitted values from logistic regression models with other possible confounders, including quintile age categories, sex, FIT, and comorbidities.

Statistical analyses were performed using the SAS software version 9.4 (SAS Institute, Cary, NC, USA). A *P*-value < 0.05 was considered to indicate statistical significance.

## Results

### Colorectal cancer mortality

From 2001 to 2010, a total of 18,816 eligible patients underwent colonoscopy. The patient characteristics at baseline are shown in Table 1. The mean age of the cohort was 61.3 years, and 12,890 (68.51%) patients were male. There were four colorectal cancer deaths during the observation period (total observed person-years, 67,119); the characteristics of these cases are shown in S1 Table. Of the four patients who died, three experienced less than 3 years between initial colonoscopy and colorectal cancer occurrence.

**Table 1:** Baseline characteristics of the patients (N = 18,816).

Factors	N = 18,816
Age category (years)	
< 50	3,499 (18.60)
50–59	4,459 (23.70)
60–69	5,382 (28.60)
70–79	4,276 (22.73)
≥ 80	1,200 (6.38)
Sex, male	12,890 (68.51)
Current smoker*	2344 (25.48)
Family history of colorectal cancer*	105 (2.95)
**Comorbidities**	
Ischemic heart diseases	2579 (13.71)
Chronic heart failure	1634 (8.68)
Peripheral vascular diseases	1074 (5.71)
Cerebral vascular diseases	1240 (6.59)
Dementia	189 (1.00)
COPD	354 (1.88)
Collagen diseases	567 (3.01)
Peptic ulcer diseases	5998 (31.88)
Diabetes mellitus	4692 (24.94)
Chronic kidney disease	304 (1.62)
Paresthesia	399 (2.12)
Leukemia	33 (0.18)
Malignant lymphoma	135 (0.72)
Liver cirrhosis	285 (1.51)
AIDS	46 (0.24)
**Indications for colonoscopy**	
Positive FIT	2995 (15.92)
**Initial colonoscopy findings**	
Polyp detection	8017 (42.61)
10-mm adenoma detection*	379 (2.80)
20-mm adenoma detection*	98 (0.73)
Therapeutic colonoscopy	3277 (17.42)
**Number of repeat colonoscopies**	
1	10705 (56.89)
2	4657 (24.75)
3	1775 (9.43)
4	721 (3.83)
5	421 (2.24)
6	279 (1.48)
7	165 (0.88)

COPD, chronic pulmonary disease; AIDS, acquired immune deficiency syndrome; FIT, fecal immunochemical test. Parentheses are percentages.

*Missing data are included.

Four, three, and one patients died from colorectal, colon, and rectal cancers, respectively; these values were significantly lower than the number of expected deaths in the general population, estimated to be 53.1, 34.0, and 19.1, respectively. The standardized all colorectal, colon, and rectal cancer mortality ratios were 0.08 (95% CI, 0.02–0.17), 0.09 (0.02–0.22), and 0.05 (0.00002–0.21), respectively. (Table 2)

**Table 2:** Observed and expected cancer mortalities and incidences during follow-up.

	Mortality			Incidence		
	Observed	Expected	SMR (95% CI)	Observed	Expected	SIR (95% CI)
**All CRCs**						
Overall	4	53.1	0.08 (0.02–0.17)	34	68.4	0.50 (0.34–0.68)
Sex						
Female	2	10.6	0.19 (0.02–0.54)	7	10.8	0.65 (0.26–1.21)
Male	2	42.5	0.05 (0.004–0.14)	27	57.5	0.47 (0.31–0.66)
**Colon cancer**						
Overall	3	34.0	0.09 (0.02–0.22)	29	42.8	0.68 (0.45–0.95)
Sex						
Female	2	7.8	0.26 (0.02–0.74)	6	7.8	0.77 (0.28–1.50)
Male	1	26.2	0.04 (0.0001–0.15)	23	35.0	0.66 (0.42–0.95)
**Rectal cancer**						
Overall	1	19.1	0.05 (0.00002–0.21)	5	25.5	0.20 (0.06–0.41)
Sex						
Female	0	2.8	NA	1	3.0	0.33 (0.0001–1.30)
Male	1	16.3	0.06 (0.00002–0.24)	4	22.5	0.18 (0.05–0.39)

CRC, colorectal cancer; SMR, standardized mortality ratio; SIR, standardized incidence ratio; CI, confidence interval; NA, not applicable.

**Fig 1:**
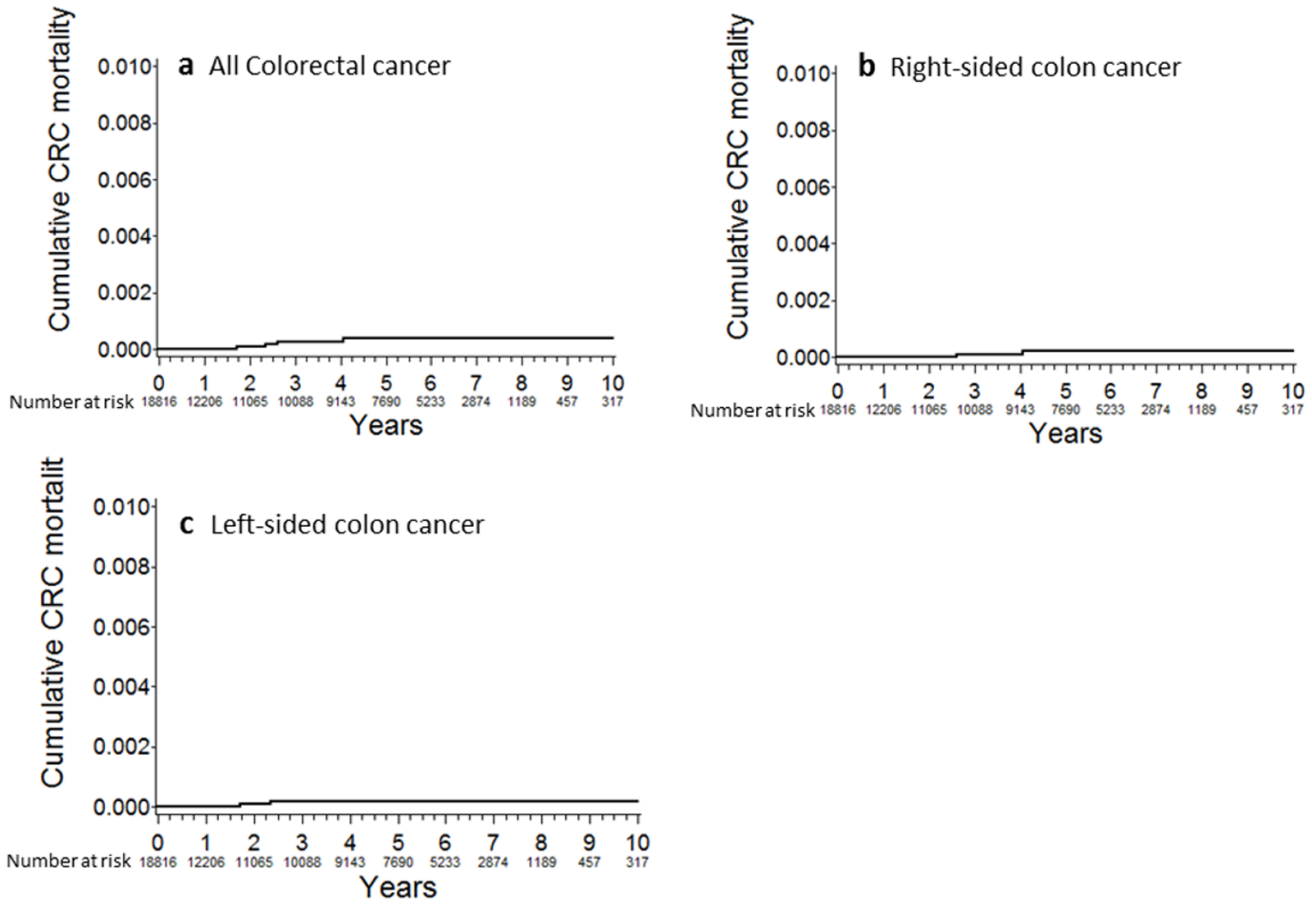
Cumulative colorectal cancer mortality. (a) All colorectal cancer; (b) Right-sided colon cancer; (c) Left-sided colon cancer.

The cumulative all colorectal cancer mortality were 0.0088% at 2 years, 0.028% at 4 years, 0.039% at 6 years, 0.039% at 8 years, and 0.039% at 10 years post-baseline (Fig 1a). The cumulative mortality of right-sided colon cancer was similar to that of left-sided colon cancer (Fig 1b and 1c).

### All-cause mortality

There were 586 deaths (3.11%) during the observation period (total observed person-years, 67,119) and the proportion of colorectal cancer-related mortality to all-cause mortality was 0.68% (4/586) in our cohort; that value was significantly lower than the number of expected deaths in the general population, estimated to be 2,703. The standardized all-cause mortality ratios were 0.22 (95% CI, 0.206–0.23).

### Colorectal cancer incidence

There were 34 new cases of colorectal cancer during the observation period (total observed person-years, 29,974). Of these, 29 patients (85.3%) could receive curative surgery (Table 3).

**Table 3:** Characteristics of patients with colorectal cancer.

Factors	N = 34
**Site of cancer at diagnosis**	
**Right-sided colon**	18 (52.94)
Cecum	6 (17.65)
Ascending	6 (17.65)
Transverse	6 (17.65)
**Left-sided colon**	16 (47.06)
Descending	1 (2.94)
Sigmoid	10 (29.41)
Rectum	5 (14.71)
**UICC cancer stage**	
1	8 (23.53)
2	16 (23.53)
3	20 (11.76)
4	21 (2.94)
Missing data	17 (38.24)
**Treatment**	
Endoscopy	6 (17.65)
Surgery	19 (55.88)
Surgery and chemotherapy	4 (11.76)
Chemotherapy	1 (2.94)
Chemoradiotherapy	1 (2.94)
Best supportive care	3 (8.82)

UICC, Union for International Cancer Control. Values in parentheses are percentages.

*Stage 1 included stage 0.

The incidences of all colorectal, colon, and rectal cancers were 34, 29, and 5 cases, respectively, in our cohort, while those expected in the general population were 68.4, 42.8, and 25.5 cases, respectively. The standardized incidence rates of all colorectal, colon, and rectal cancer were 0.50 (95% CI 0.348–0.68), 0.68 (0.51–0.95), and 0.18 (0.06–0.41), respectively (Table 2).

**Fig 2:**
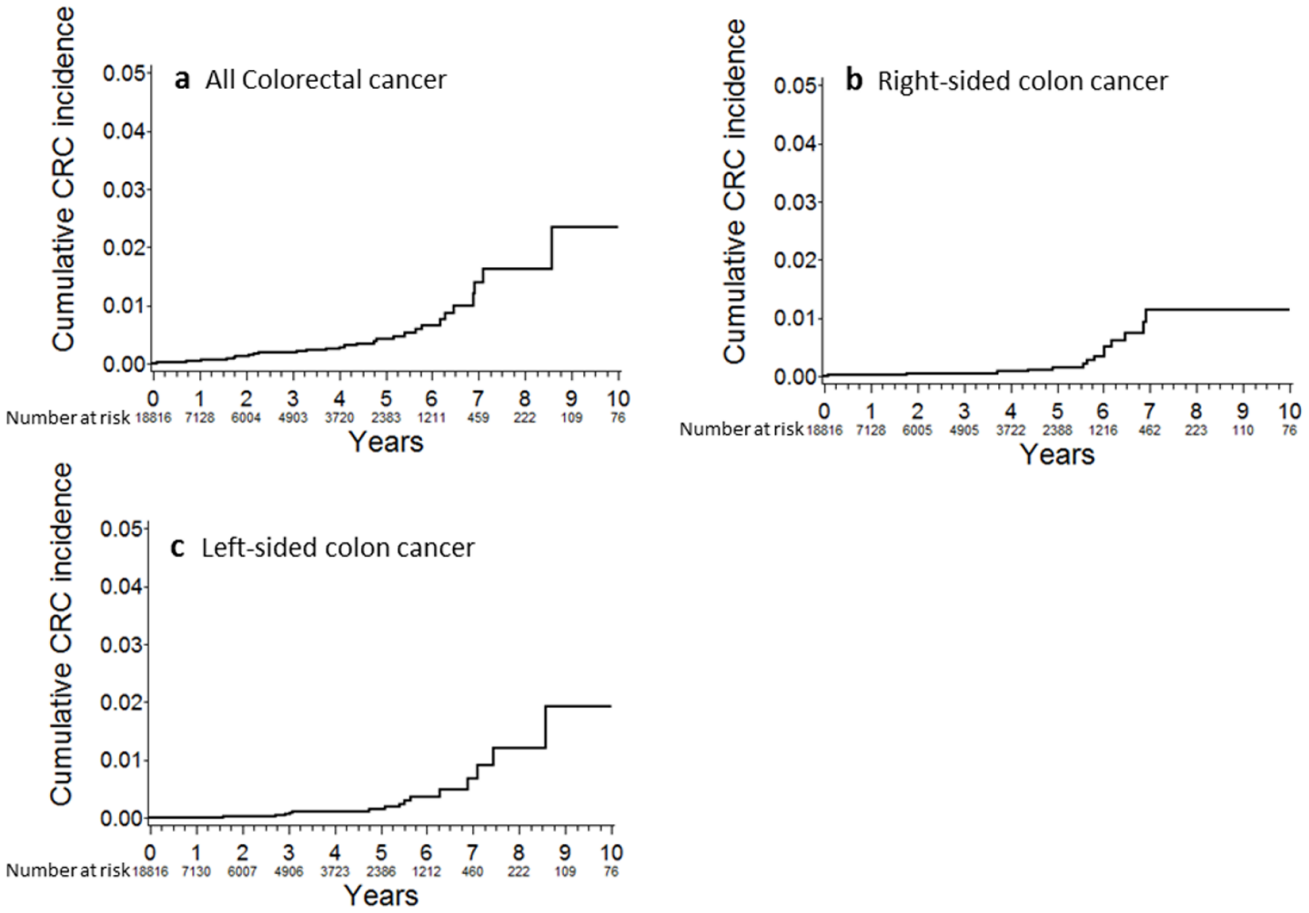
Cumulative colorectal cancer incidence. (a) All colorectal cancer; (b) Right-sided colon cancer; (c) Left-sided colon cancer.

The cumulative incidence rates of all colorectal cancer were 0.128% at 2 years, 0.265% at 4 years, 0.660% at 6 years, 1.638% at 8 years, and 2.351% at 10 years post-baseline (Fig 2a). The cumulative incidence of right-sided colon cancer was similar to that of left-sided colon cancer (Fig 2b and 2c).

### Factors associated with colorectal cancer mortality and incidence

The factors associated with mortality and incidence of colorectal cancer are shown in Table 4 and S2 Table. All colorectal cancer deaths occurred in patients aged > 70 years. Peripheral vascular disease and chronic obstructive pulmonary disease (COPD) were associated with an increased risk of colorectal cancer mortality.

**Table 4:** Factors associated with colorectal cancer mortality (N = 18,816).

Factors	No. of deaths	1,000 person- years	Crude HR	*P*-value
Age category				
< 50	0	0	1	
50–59	0	0	NA	NA
60–69	0	0	NA	NA
70–79	2	0.123	NA	NA
≥ 80	2	0.580	NA	NA
Sex				
Female	2	0.105	1	
Male	2	0.042	0.41 (0.06–2.92)	0.374
Current smoker	0	0	NA	NA
Family history of CRC	0	0	NA	NA
**Comorbidities**				
Ischemic heart diseases	2	0.135	3.65 (0.51–25.9)	0.196
Chronic heart failure	2	0.208	6.13 (0.86–43.6)	0.070
Peripheral vascular diseases	2	0.290	9.59 (1.35–68.1)	**0.024**
Cerebral vascular diseases	1	0.147	2.99 (0.31–28.7)	0.344
Dementia	0	0	NA	NA
COPD	1	0.502	11.1 (1.15–106.5)	**0.037**
Collagen diseases	0	0	NA	NA
Peptic ulcer diseases	3	0.094	3.39 (0.35–32.6)	0.291
Diabetes mellitus	3	0.109	4.66 (0.48–44.8)	0.183
Chronic kidney diseases	0	0	NA	NA
Paresthesia	0	0	NA	NA
Leukemia	0	0	NA	NA
Malignant lymphoma	0	0	NA	NA
Liver cirrhosis	0	0	NA	NA
AIDS	0	0	NA	NA
**Indication for colonoscopy**				
Positive FIT	1	0.060	1.00 (0.10–9.60)	0.999
**Initial colonoscopy findings**				
Polyp detection	2	0.064	1.14 (0.16–8.07)	0.898
10-mm adenoma detection^†^	0	0	NA	NA
20-mm adenoma detection^†^	0	0	NA	NA
Therapeutic colonoscopies	0	0	NA	NA
**Number of colonoscopies**				
1	0	0	NA	NA
2	3	0.130	1	
3	1	0.106	1.60 (0.17–15.3)	0.686
4	0	0	NA	NA
5	0	0	NA	NA
6	0	0	NA	NA
7	0	0	NA	NA

^†^The crude and adjusted hazard ratios of 10-mm and 20-mm adenoma detection were calculated using imputation data.

CRC, colorectal cancer; COPD, chronic pulmonary disease; AIDS, acquired immune deficiency syndrome; FIT, fecal immunochemical test; HR, hazard ratio; NA, not applicable.

Comorbidities including chronic heart failure, COPD, dementia, diabetes mellitus, chronic kidney disease, malignant lymphoma, and 10--mm adenoma detection were also associated with an increased risk of colorectal cancer incidence. No significant differences were observed between an increased number of colonoscopy procedures and both colorectal cancer mortality and incidence.

## Discussion

In this large, multicenter, colonoscopy cohort study, we found that the colorectal cancer mortality rate, including that of proximal colon cancer, in patients who received colonoscopy without diagnosis of colorectal cancer was significantly decreased compared with the mortality rate of the general population. Additionally, the colorectal cancer incidence decreased in patients who received colonoscopy.

Our study is the first to show, using colonoscopy-based cohort data, that colonoscopy prevents death from and occurrence of colorectal cancer. Although our study excluded colorectal cancer cases within 6 month of colonoscopy as applied by many previous studies, our data showed the greatest mortality reduction (standardized mortality ratio, 0.08) and the lowest colorectal cancer mortality (0.06 deaths per 1,000 person-years).[13,14,16,24] Several previous studies that used secondary data reported the effectiveness of colonoscopy for reducing colorectal cancer mortality (HR 0.32–0.71 and 0.27–1.0 deaths per 1,000 person-years).[13–17,20,21] For example, analyzing health care payment database and excluding index colonoscopies associated with colorectal cancer diagnosis within 6 month, Singh et al. reported 1.0 deaths per 1,000 person-years after colonoscopy and 29% reduction in CRC mortality compared to general population.[17]

One of the possible reasons which account for low mortality in ours is the study period evaluated, as the majority of previous studies evaluated the effects of colonoscopies performed in the 1980s and 1990s; however, we included patients who received colonoscopies between 2001 and 2010. Owing to improvements in endoscopy itself as well as refinement of the insertion technique and bowel preparation, the diagnostic yield of colonoscopy was undoubtedly better in our study, which might lead to fewer cases of missed colorectal cancer and to better prognosis. Another possibility is that relatively short follow-up periods. Some patients may have been diagnosed as colorectal cancer elsewhere and may have been dead without indentified in this study. However we also evaluated all-cause mortality as well. The proportion of colorectal cancer-related mortality to all-cause mortality was 0.68% (4/586) in our cohort, which is significantly lower than 2.0% (53.1/2703) which is expected in general population or 2.06% (26,164/1273,020) of actual death in Japanese population in 2014.[1] Indeed, most of participating institutes for this study are high-volume medical centers that provide almost all medical services. As we have reported previously, the incidence of post-colonoscopy colorectal cancer is low in patients who have received a colonoscopy at a high-volume referral center in Japan.[27] Thus, the observed lower mortality is partly due to the high quality of the colonoscopies at the study hospitals, compared with general hospitals in Japan. In addition, our patients may have better accessibility to large medical centers or high health awareness, which may account for the lower all-cause mortality in the study participants.

In our study, colonoscopy reduced both right- and left-sided colon cancer mortality and incidence, which is inconsistent with results from several studies.[23,28] One possible explanation for this is the difference in the quality of colonoscopic examination, as described above. In addition, colonoscopies were performed by gastroenterologists at specialized institutions in our study, and we did not include patients who received colonoscopies by general practitioners. However, only 6–31% of colonoscopies in previous studies were performed by gastroenterologists.[13,15,23] Colonoscopies performed by general practitioners are associated with an increased risk of missed colorectal cancer cases compared with colonoscopies performed by gastroenterologists.[28,29] This difference in quality may be associated with the difference in colorectal cancer mortality rates between right- and left-sided colon cancers. The protective effect for right-sided colorectal cancer mortality has also been reported in recent studies.[17,18]

In our study, therapeutic colonoscopy was not significantly associated with a decreased risk of death from or incidence of colorectal cancer. This result was inconsistent with several previous studies, which showed reduced colorectal cancer mortality associated with therapeutic colonoscopy.[17,21] Several possibilities could explain this inconsistency. First, there is an inconsistency regarding the magnitude of colonoscopy surveillance. Zauber et al. investigated the National Polyp Study cohort and reported effective cancer-related death reduction by polypectomy.[21] However, the majority of these cohorts received surveillance colonoscopy after polypectomy, which could reduce interval colorectal cancer and death. Next, the majority of therapeutic colonoscopies in our study were performed in patients with small polyps; thus, the effectiveness of therapeutic colonoscopy may be underestimated in our study. Lastly, our follow-up period was short compared with those of previous studies,[13–17,20,23,30] and it may have been insufficient to detect the preventive effect of polypectomy.

Our study has several strengths. First, we performed colonoscopy in all patients and collected and analyzed all colonoscopy records from the study patients, which provide extremely accurate information concerning colorectal cancer diagnosis as well as initial negative condition. Second, our estimated standardized colorectal cancer mortality and incidence rates may be more reliable than those of previous studies, because we used high-quality cancer mortality data from a database established in 1958, which covers the entire Japanese population, and revised the data annually.[26]

However, our study also has several limitations. The follow-up period was relatively short compared with those of previous studies.[13–17,20,23,30] Although over 7,000 patients were included in the mortality analysis 5 years after the initial colonoscopy (Fig 1a), further long-term investigations are needed to evaluate colorectal cancer mortality more accurately. Second, standardized mortality and incidence were estimated according to all colorectal, colon, and rectal cancers, and not right-sided or left-sided colon cancer, because the Japanese National Cancer database does not provide these data. Third, our cohort was potential biased due to left truncation by identifying and including only patients who had survived to receive colonoscopy-based surveillance; thus, we may have underestimated the colorectal cancer mortality rate and overestimate the SMR but the magnitude is unknown from these data. Finally, the generalizability of our results to patients receiving colonoscopies in low volume medical centers is uncertain because our data were largely based on high volume medical centers.

In conclusion, compared with the general population, patients who received a colonoscopy had a significantly decreased risk of colorectal cancer mortality and incidence for at least 5 years.

## Supporting information

S1 Fig Flow chart of the study population selection. (TIF)

S1 Table Characteristics of the four patients who died from colorectal cancer. (DOCX)

S2 Table Factors associated with colorectal cancer incidence (N = 18,816). (DOCX)

S1 Appendix STROBE STATEMENT checklist of items that should be included in reports of Observational Studies. (DOCX)
