# Supplementary figures and images for: Colonoscopy reduces colorectal cancer mortality: A multicenter, long-term, colonoscopy-based cohort study
Source: PLoS One. 2017 Sep 28;12(9):e0185294. doi: 10.1371/journal.pone.0185294 (PMC5619740; doi:10.1371/journal.pone.0185294)

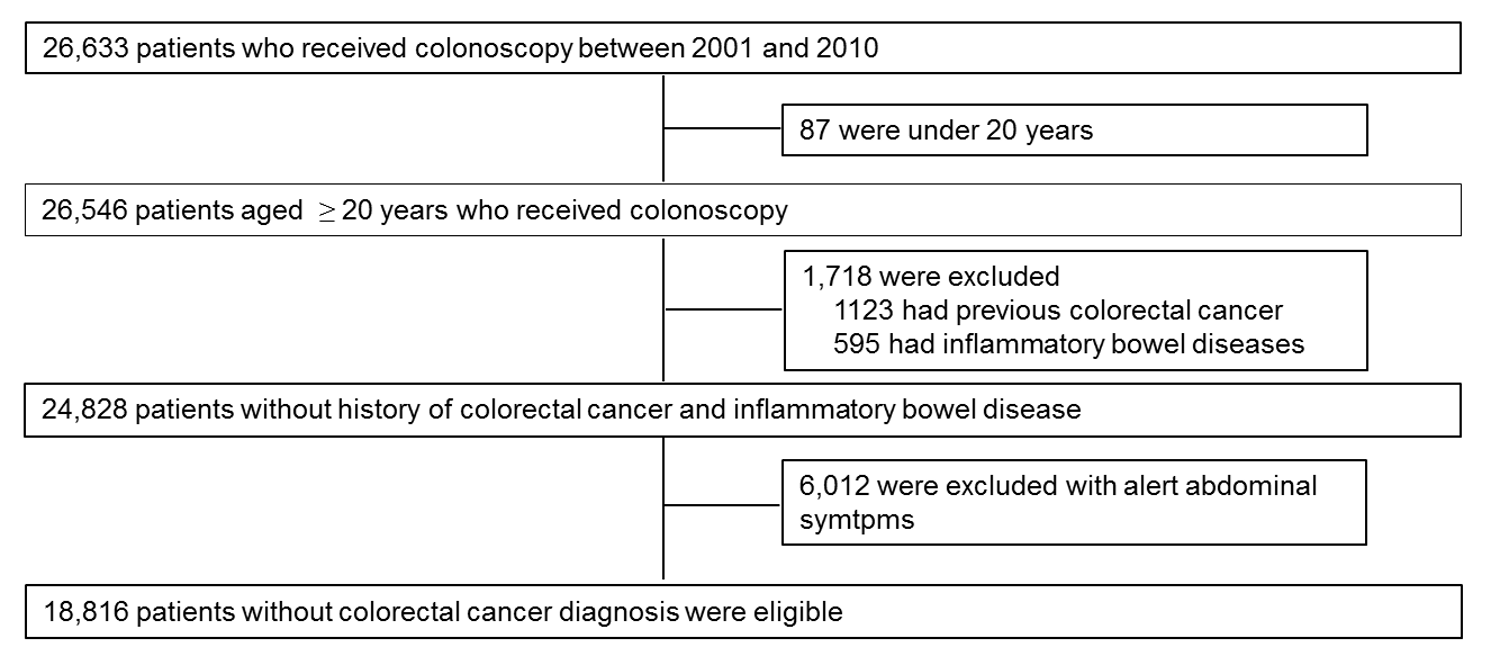

Supplement: S1 Fig — (TIF) [file pone.0185294.s001.tif]
