# Supplementary material for: Colonoscopy reduces colorectal cancer mortality: A multicenter, long-term, colonoscopy-based cohort study
Source: PLoS One. 2017 Sep 28;12(9):e0185294. doi: 10.1371/journal.pone.0185294 (PMC5619740; doi:10.1371/journal.pone.0185294)
Supplement: S1 Table — (DOCX) [file pone.0185294.s002.docx]

# **S1 Table. Characteristics of the four patients who died from colorectal cancer.**

| No. | Age | Sex | Initial CS | | Interval to CRC incidence* | CRC findings | | | Interval to CRC death** | Total no. of CS |
| --- | --- | --- | --- | --- | --- | --- | --- | --- | --- | --- |
|  |  |  | 10-mm adenoma | 20-mm adenoma |  | Site | UICC stage | Treatment |  |  |
| 1 | 83 | M | No | Yes | 6.5 | S | 4 | Surgery + chemotherapy | 7.0 | 2 |
| 2 | 80 | M | No | No | 3.7 | R | 4 | Surgery | 3.8 | 3 |
| 3 | 75 | M | No | No | 4.0 | T | 3 | Surgery | 4.1 | 2 |
| 4 | 72 | M | No | No | 1.2 | R | 3 | Surgery + chemotherapy | 2.4 | 3 |

M, male; F, female; CRC, colorectal cancer; UICC, Union for International Cancer Control; C, cecum; T, transverse; D, descending, S, sigmoid; R, rectum; CS, colonoscopy.

* Interval between initial CS and CRC incidence ** Interval between initial CS and CRC death
