# Supplementary material for: Colonoscopy reduces colorectal cancer mortality: A multicenter, long-term, colonoscopy-based cohort study
Source: PLoS One. 2017 Sep 28;12(9):e0185294. doi: 10.1371/journal.pone.0185294 (PMC5619740; doi:10.1371/journal.pone.0185294)
Supplement: S2 Table — (DOCX) [file pone.0185294.s003.docx]

# **S2 Table. Factors associated with colorectal cancer incidence (N = 18,816).**

| Factors | No. of cases | 1,000 person-years | Crude HR | *P*-value |
| --- | --- | --- | --- | --- |
| Age category |  |  |  |  |
| < 50 | 1 | 0.270 | 1 |  |
| 50–59 | 10 | 1.261 | 4.40 (0.56–34.4) | 0.157 |
| 60–69 | 8 | 0.777 | 2.85 (0.36–22.8) | 0.320 |
| 70–79 | 10 | 1.429 | 6.09 (0.78–47.6) | 0.084 |
| ≥ 80 | 5 | 4.791 | 25.3 (2.93–219) | **0.003** |
| Sex  Female | 7 | 0.948 | 1 |  |
| Male | 27 | 1.195 | 1.06 (0.46–2.45) | 0.892 |
| Current smoker^†^ | 2 | 0.489 | 0.58 (0.17–1.95) | 0.382 |
| Family history of CRC^†^ | 1 | 4.208 | 3.54 (0.36–35.2) | 0.281 |
| **Comorbidities** |  |  |  |  |
| Ischemic heart diseases | 8 | 1.368 | 1.18 (0.54–2.62) | 0.677 |
| Chronic heart failure | 10 | 2.452 | 2.53 (1.21–5.29) | **0.014** |
| Peripheral vascular diseases | 6 | 2.260 | 1.71 (0.70–4.18) | 0.242 |
| Cerebral vascular diseases | 4 | 1.663 | 1.46 (0.51–4.15) | 0.478 |
| Dementia | 2 | 5.583 | 5.63 (1.35–23.6) | **0.018** |
| COPD | 3 | 2.991 | 2.65 (0.81–8.67) | 0.108 |
| Collagen diseases | 3 | 2.119 | 1.91 (0.58–6.24) | 0.286 |
| Peptic ulcer diseases | 20 | 1.426 | 1.48 (0.74–2.93) | 0.267 |
| Diabetes mellitus | 211 | 1.827 | 2.15 (1.07–4.34) | **0.033** |
| Chronic kidney diseases | 3 | 4.609 | 4.09 (1.25–13.4) | **0.020** |
| Paresthesia | 0 | 0 | NA | NA |
| Leukemia | 0 | 0 | NA | NA |
| Malignant lymphoma | 2 | 6.443 | 7.28 (1.73–30.6) | **0.007** |
| Liver cirrhosis | 0 | 0 | NA | NA |
| AIDS | 0 | 0 | NA | NA |
| **Indication of colonoscopy** |  |  |  |  |
| Positive FIT | 12 | 1.624 | 1.78 (0.88–3.60) | 0.111 |
| **Initial colonoscopy findings** |  |  |  |  |
| Polyp detection | 19 | 1.175 | 1.18 (0.60–2.32) | 0.637 |
| 10-mm adenoma detection^†^ | 3 | 4.781 | 5.10 (1.62–16.1) | **0.005** |
| 20-mm adenoma detection^†^ | 0 | 0 | NA | NA |
| Therapeutic colonoscopies | 6 | 0.934 | 0.83 (0.34–1.99) | 0.669 |
| **Number of colonoscopies** |  |  |  |  |
| 1 | 0 | 0 | NA | NA |
| 2 | 15 | 1.065 | 1 |  |
| 3 | 8 | 1.132 | 1.19 (0.50–2.81) | 0.697 |
| 4 | 7 | 2.026 | 1.79 (0.73–4.40) | 0.207 |
| 5 | 2 | 0.883 | 0.65 (0.15–2.90) | 0.570 |
| 6 | 0 | 0 | NA | NA |
| 7 | 2 | 2.103 | 1.70 (0.39–7.48) | 0.486 |

^†^The crude and adjusted hazard ratios for current smoking status, family history of colorectal cancer, and 10-mm and 20-mm adenoma detection were calculated using imputation data. CRC, colorectal cancer; COPD, chronic pulmonary disease; AIDS, acquired immune deficiency syndrome; FIT, fecal immunochemical test; HR, hazard ratio; NA, not applicable.
